# Supplementary material for: Chemokine-Binding All-D-CLIPS Peptides Identified Using Mirror-Image Phage Display
Source: ACS Chem Biol. 2025 Oct 29;20(11):2798–807. doi: 10.1021/acschembio.5c00726 (PMC12645432; doi:10.1021/acschembio.5c00726)
Supplement: Supplementary file 1 [file cb5c00726_si_001.pdf]

Chemokine-binding all-D-CLIPS™ peptides identified using mirror-image phage display

Stepan S. Denisov[1,2*], Emilia L. Bialek[2,3], Fabio Beretta[3], Gintare Smagurauskaite[4], Hans Ippel[2], Eline Fijlstra[5], Sangram S. Kale[5], Peter Timmerman[5], Tilman M. Hackeng[2], Paul Proost[3], Michael Goldflam[†,5], Ingrid Dijkgraaf[†,2]

[1]Insitute of Biological Chemistry, University of Vienna, Währinger Str. 38, 1090 Vienna, Austria
[2] Department of Biochemistry, Maastricht University, Cardiovascular Research Institute Maastricht (CARIM), Universiteitssingel 50, 6229 ER, Maastricht, the Netherlands.
[3]Rega Institute, KU Leuven, Herestraat 49, 3000, Leuven, Belgium
[4]RDM Cardiovascular Medicine, University of Oxford, Roosevelt Drive, Oxford OX3 7BN, UK
[5]Biosynth B.V., Zuidersluisweg 2, 8243 RC Lelystad, the Netherlands.
† Authors contributed equally.
Correspondence: stepan.denisov@univie.ac.at

Table of Contents

Table S1. Secondary chemical shifts and CSI 3.0 algorithm output for D-2A5 and D-3A11....3

Figure S1. LC-MS analysis of thz^{34}-leu^{49}-MPA-Leu 1....4

Figure S2. LC-MS analysis of cys^{50}-ser^{72}-Lys(Gly^{2}-biotin) 2....5

Figure S3. LC-MS analysis of ser^{1}-his^{33}-MPA-Leu 3....6

Figure S4. LC-MS analysis of NCL of 1 and 2....7

Figure S5. LC-MS analysis of cys^{34}-ser^{72}-Lys(Gly$_2$-biotin) 4....8

Figure S6. LC-MS analysis of NCL of 4 and 3....9

Figure S7. LC-MS analysis of reduced D-CXCL8-KGG-biotin 5....10

Figure S8. D-CXCL8-biotin refolding....11

Figure S9. Crude CLIPS™ L-peptide interaction with D-CXCL8-KGG-biotin (6)....12

Figure S10. LC-MS analysis of L-CLIPS™ peptide 3A11....13

Figure S11. LC-MS analysis of L-CLIPS™ peptide 2A5....14

Figure S12. Synthesis of L-CLIPS™ peptide 1H4....15

Figure S13. LC-MS analysis of L-CLIPS™ peptide 3A12....16

Figure S14. LC-MS analysis of D-CLIPS™ peptide 1H4....17

Figure S15. LC-MS analysis of D-CLIPS™ peptide 2A5....18

Figure S16. LC-MS analysis of D-CLIPS™ peptide 3A11....19

Figure S17. LC-MS analysis of D-CLIPS™ peptide 3A12....20

Figure S18. Binding of D-peptides to L- and D-CXCL8....21

Figure S19. Binding of D-2A5 to L-CCL5 and L-CXCL4....22

Figure S20. Far-UV CD spectra of L-CLIPS peptides....23

Figure S21. Secondary structure composition of CLIPSTM peptides determined by the BeStSel algorithm using CD spectra at 0.1 mg/mL of L-peptides. **24**

Figure S22. Ramachandran plots of calculated D-peptide models **25**

Figure S23. ^{15}N-^{1}H HSQC spectra [^{15}N, ^{13}C] L-CXCL8 dimer **26**

Figure S24. ^{15}N-^{1}H HSQC spectra [^{15}N, ^{13}C] L-CXCL8/D-2A5 complex. **27**

Figure S25. ^{15}N-^{1}H HSQC spectra of [^{15}N, 13C] L-CXCL8/D-3A11 complex **28**

Table S1. Secondary chemical shifts and CSI 3.0 algorithm output for D-2A5 and D-3A11.
B-prob, C-prob, and H-prob stand for the probability of β-sheet, coil, and α-helix, respectively.

D-2A5

Seq	Cα	Cβ	H	Hα	B-prob	C-prob	H-prob
A1	51.61	20.04	8.04	3.883	0.14	0.86	0.00
C2		35.22	8.686	5.054	0.86	0.13	0.01
W3	56.15	31.65	8.338	4.961	0.93	0.06	0.00
V4	60.75	34.20	8.58	4.807	0.98	0.02	0.00
I5	59.58	40.50	8.425	4.544	0.92	0.08	0.00
S6		64.65	8.429	5.019	0.77	0.23	0.00
Y7	58.62	39.73	8.742	4.42	0.37	0.51	0.12
D8	53.90		8.762	4.128	0.18	0.82	0.00
G9	45.88		7.838		0.08	0.85	0.07
Y10	57.34	39.88	7.692	4.556	0.34	0.65	0.01
E11	55.11	30.90	8.51	4.81	0.86	0.14	0.00
Y12	57.12	40.86	8.588	4.692	0.83	0.17	0.00
C13		35.08	8.714	4.914	0.80	0.17	0.03
G14	44.63		8.209		0.14	0.86	0.01

D-3A11

Seq	Cα	Cβ	H	Hα	B-prob	C-prob	H-prob
A1	51.34	20.82		4.066	0.37	0.63	0.00
C2	55.30	36.02	8.56	4.692	0.79	0.19	0.02
F3	56.81	42.00	8.66	5.092	0.94	0.06	0.00
L4	54.99	45.53	8.536	4.491	0.85	0.15	0.00
A5	51.07	21.14	8.355	4.813	0.92	0.08	0.00
M6	55.05	35.28	8.601	4.571	0.70	0.29	0.01
D7	53.41	39.23	8.97	4.539	0.09	0.91	0.00
G8	45.15		8.495		0.12	0.86	0.02
V9	61.27	34.69	7.784	4.147	0.69	0.31	0.00
E10	54.88	30.58	8.242	4.622	0.63	0.37	0.00
Y11	56.89	41.31	8.71	4.681	0.88	0.12	0.00
R12	55.00	32.72	8.5	4.515	0.71	0.29	0.00
C13	55.93	35.60	8.556	4.676	0.77	0.20	0.02
G14	44.59		8.568		0.15	0.84	0.01

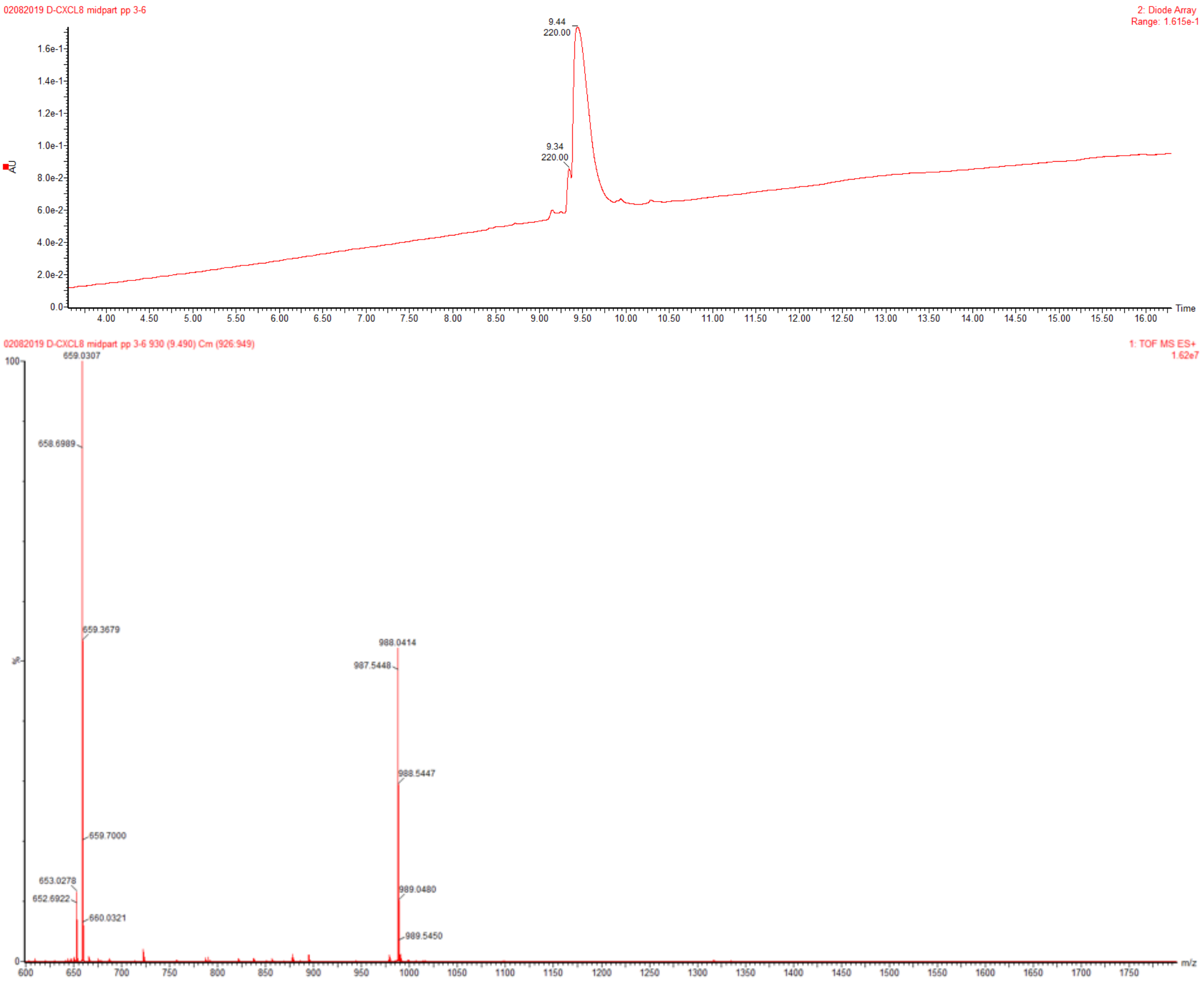

Figure S1. LC-MS analysis of thz^{34}-leu^{49}-MPA-Leu 1.
The HPLC trace (top) and ESI mass spectrum (bottom) of 1. Observed deconvoluted $[M+H]^+$ MW = 1974.1 Da, calculated $[M+H]^+$ MW = 1973.9 Da.

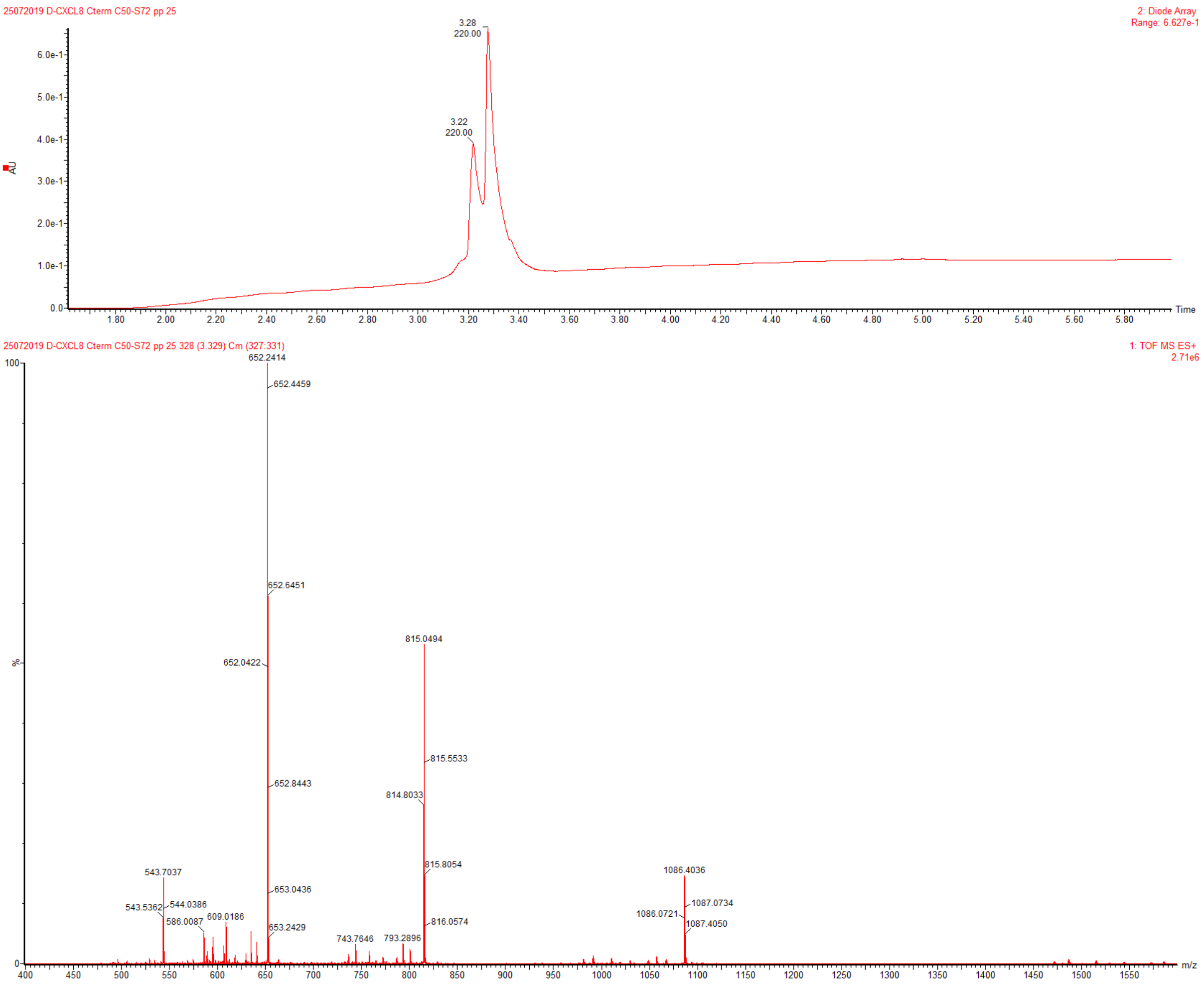

Figure S2. LC-MS analysis of cys^{50}-ser^{72}-Lys(Gly^{2}-biotin) 2.

The HPLC trace (top) and ESI mass spectrum (bottom) of 2. Observed deconvoluted $[M+H]^+$ MW = - 3256.2 Da, calculated $[M+H]^+$ MW = 3255.9 Da. The minor peak is a Glu deletion.

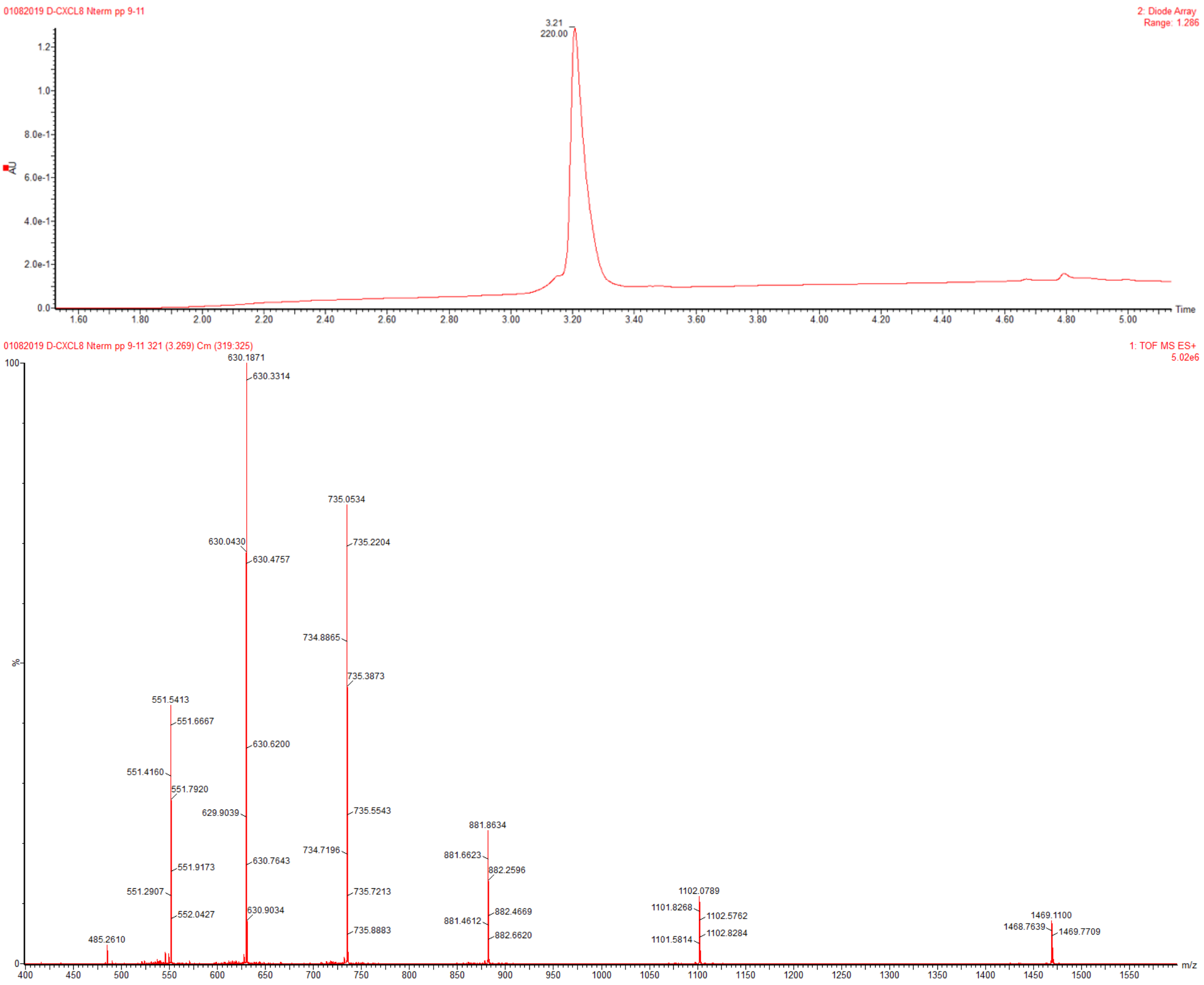

Figure S3. LC-MS analysis of ser^{1}-his^{33}-MPA-Leu 3.
The HPLC trace (top) and ESI mass spectrum (bottom) of 3. Observed deconvoluted $[M+H]^+$ MW = 4403.3 Da, calculated $[M+H]^+$ MW = 4403.2 Da.

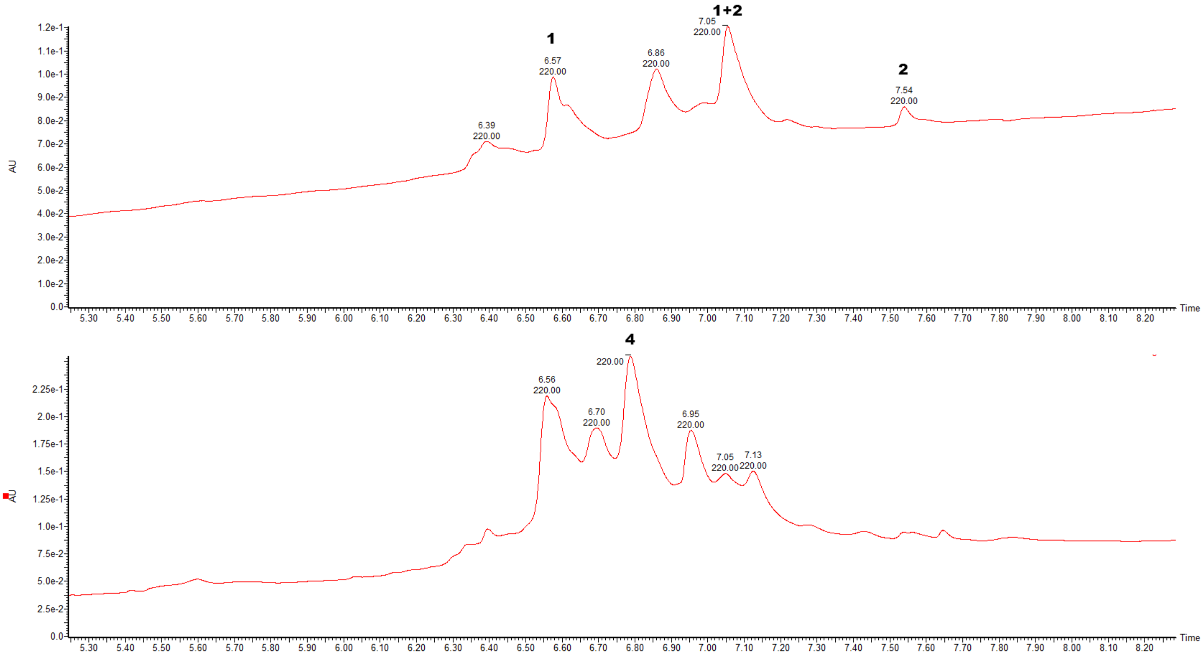

Figure S4. LC-MS analysis of NCL of 1 and 2.
The HPLC traces of the ligation mixture at t = 2 h (top) and after overnight deprotection in 6 M Gdn-HCl, 0.1 M N-methylhydroxylamine, 0.1 M acetate pH 4 (bottom). The NCL product is designated as **1+2**.

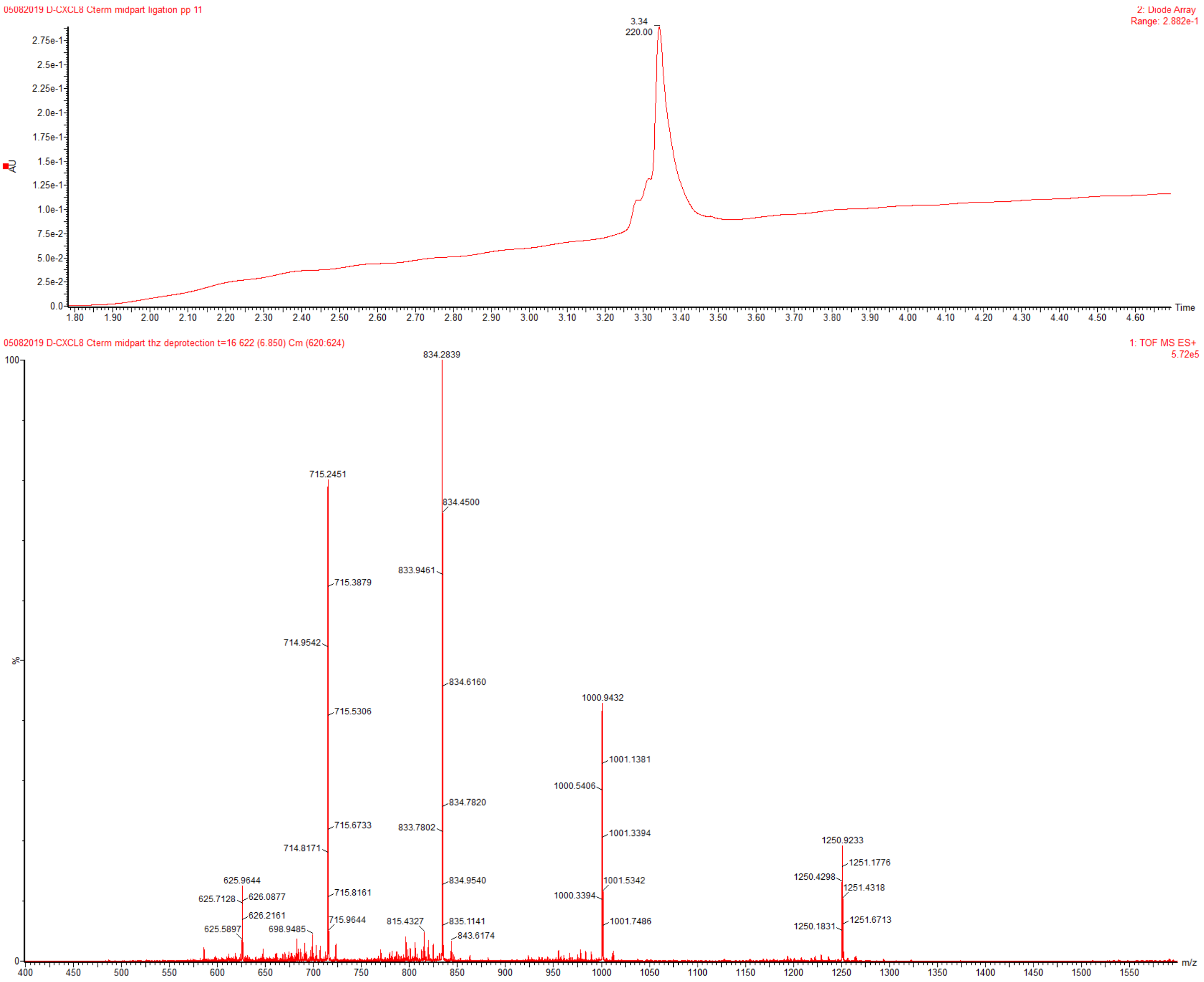

Figure S5. LC-MS analysis of cys[34]-ser[72]-Lys(Gly$_2$-biotin) 4.
The HPLC trace (top) and ESI mass spectrum (bottom) of 4. Observed deconvoluted $[M+H]^+$ MW = 4997.6 Da, calculated $[M+H]^+$ MW = 4997.7 Da.

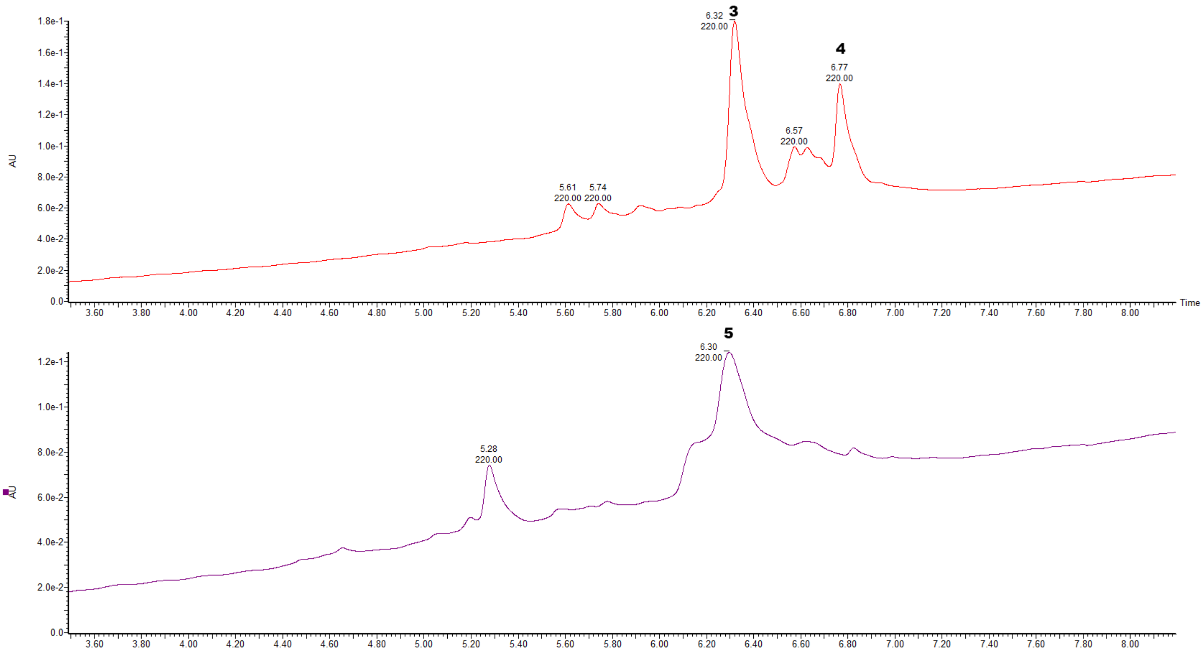

Figure S6. LC-MS analysis of NCL of 4 and 3.
The HPLC traces of the ligation mixture at t = 0 (top) and after 2 h (bottom).

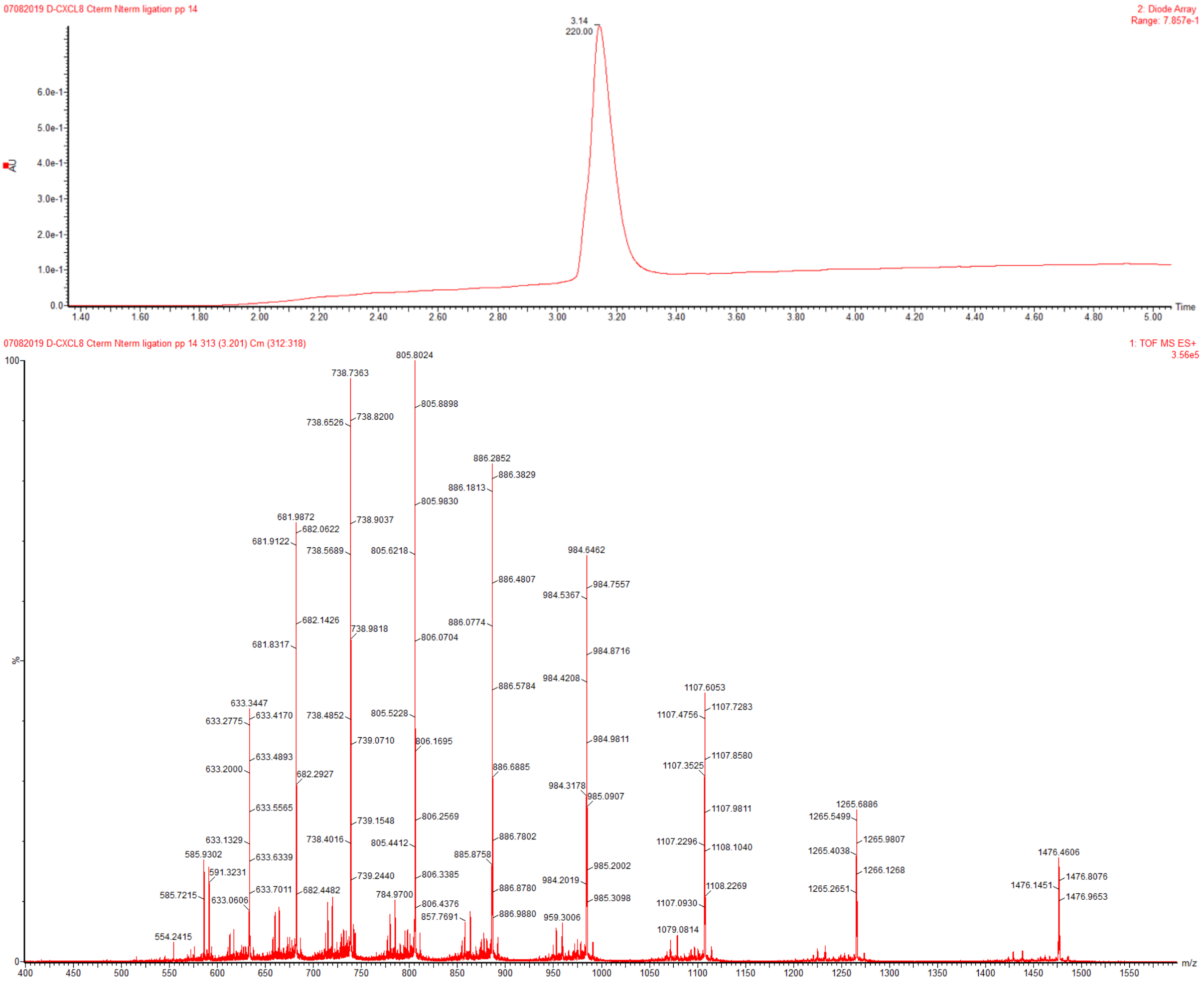

Figure S7. LC-MS analysis of reduced D-CXCL8-KGG-biotin 5.

The HPLC trace (top) and ESI mass spectrum (bottom) of 3. Observed deconvoluted $[M+H]^+$ MW = 8848.8 Da, calculated $[M+H]^+$ MW = 8848.8 Da.

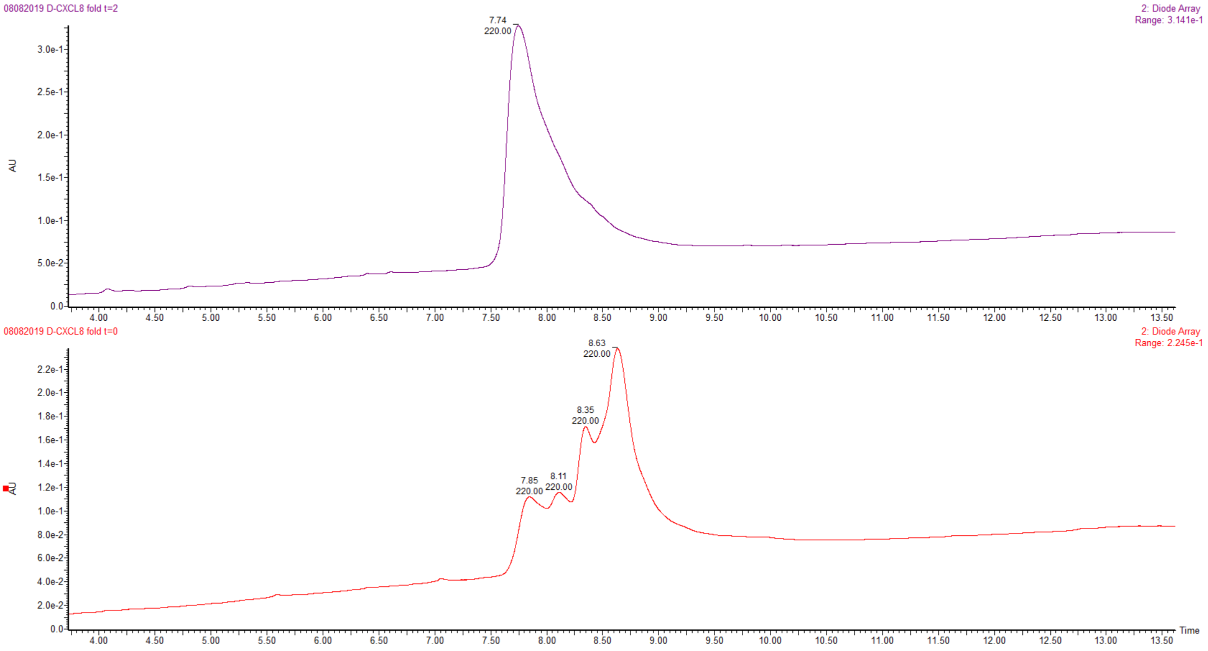

Figure S8. D-CXCL8-biotin refolding

HPLC traces of D-CXCL8-biotin under oxidative refolding conditions at t = 0 (bottom) and after 2 hours (top).

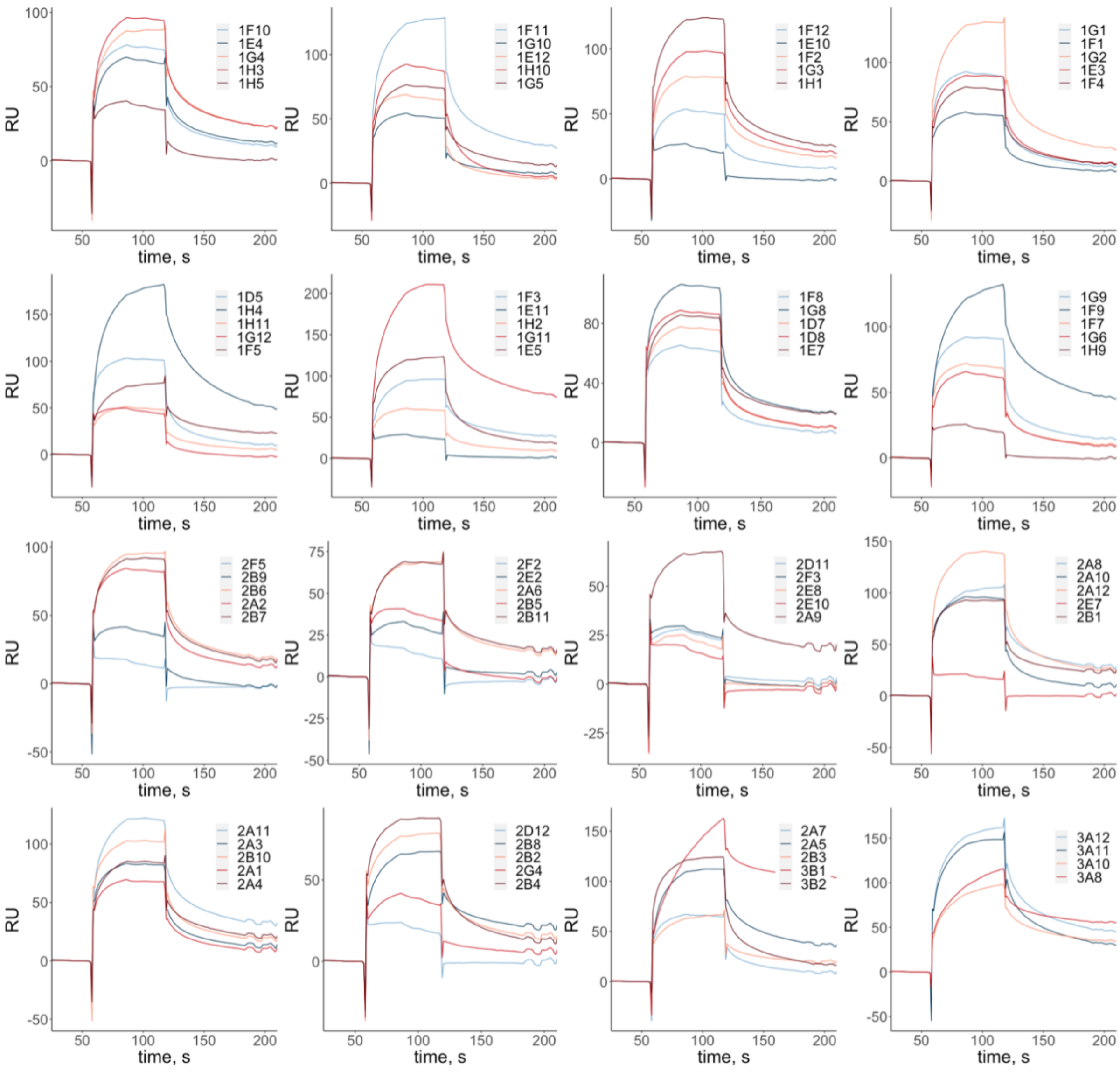

Figure S9. Crude CLIPS^{TM} L-peptide interaction with D-CXCL8-KGG-biotin (6). SPR biosensor analysis of interaction of 10 μM crude L-CLIPS^{TM} peptides with D-CXCL8-KGG-biotin (**6**).

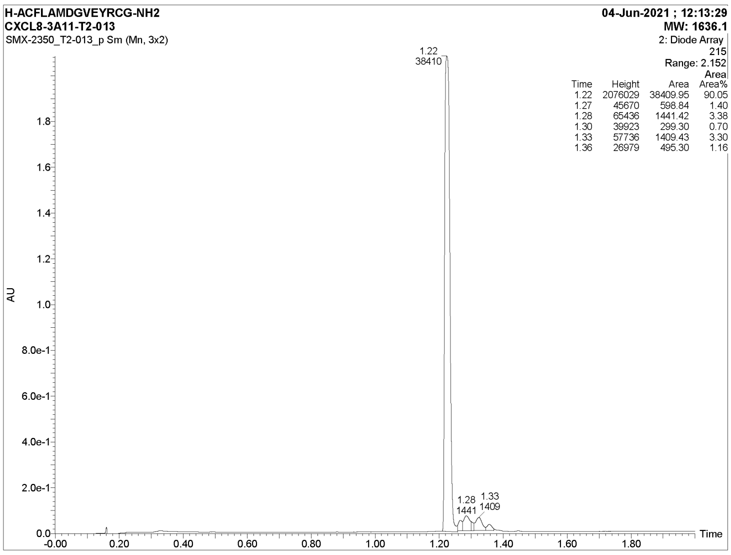

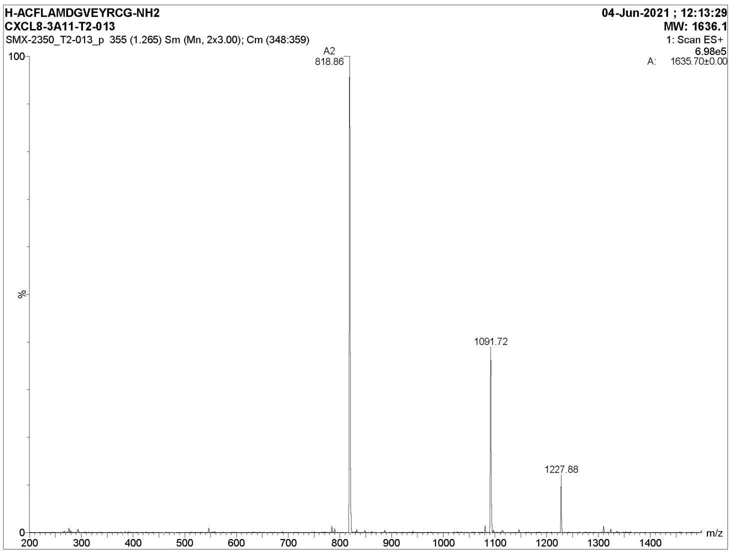

Figure S10. LC-MS analysis of L-CLIPS^{TM} peptide 3A11

The HPLC trace (top) and ESI mass spectrum (bottom) of L-CLIPS^{TM} peptide 3A11. Observed *m/z* signals: $[M+2H]^{2+}$ - 818.9, $[2M+3H]^{3+}$ - 1091.7, $[3M+4H]^{4+}$ - 1227.9; calculated *m/z* signals: $[M+2H]^{2+}$ - 819.1, $[2M+3H]^{3+}$ - 1091.7, $[3M+4H]^{4+}$ - 1228.1.

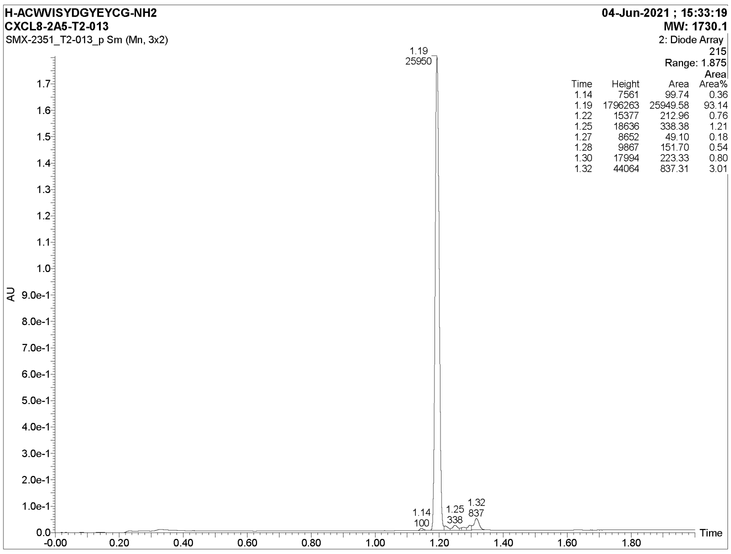

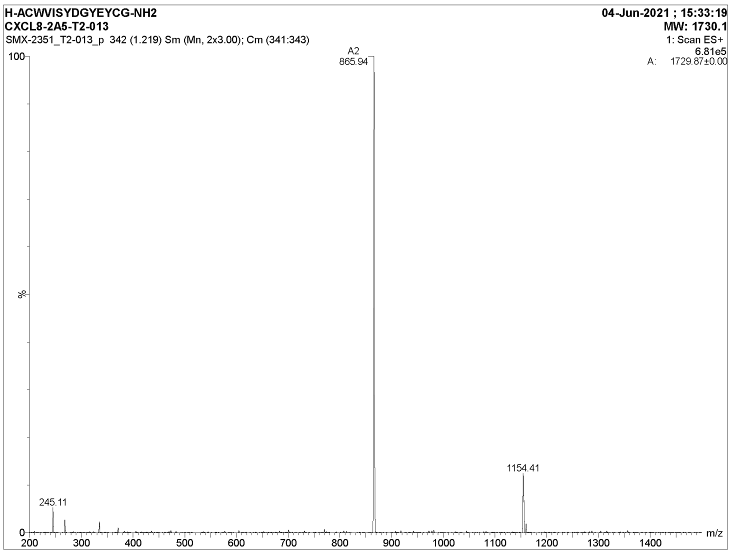

Figure S11. LC-MS analysis of L-CLIPS™ peptide 2A5.
The HPLC trace (top) and ESI mass spectrum (bottom) of L-CLIPS™ peptide 2A5. Observed *m/z* signals: $[M+2H]^{2+}$ - 865.9, $[2M+3H]^{3+}$ - 1154.4, calculated *m/z* signals: $[M+2H]^{2+}$ - 866.1, $[2M+3H]^{3+}$ - 1154.4.

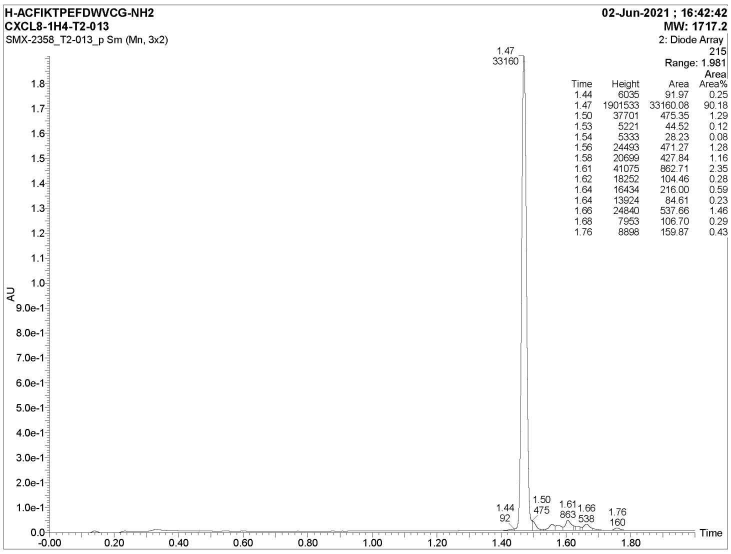

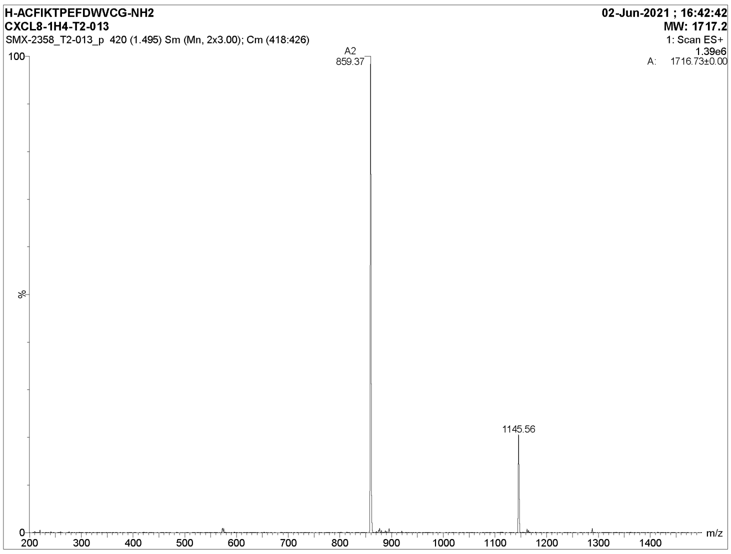

Figure S12. Synthesis of L-CLIPS™ peptide 1H4.

The HPLC trace (top) and ESI mass spectrum (bottom) of L-CLIPS™ peptide 1H4. Observed *m/z* signals: $[M+2H]^{2+}$ - 859.4, $[2M+3H]^{3+}$ - 1145.6, calculated *m/z* signals: $[M+2H]^{2+}$ - 859.6, $[2M+3H]^{3+}$ - 1145.8.

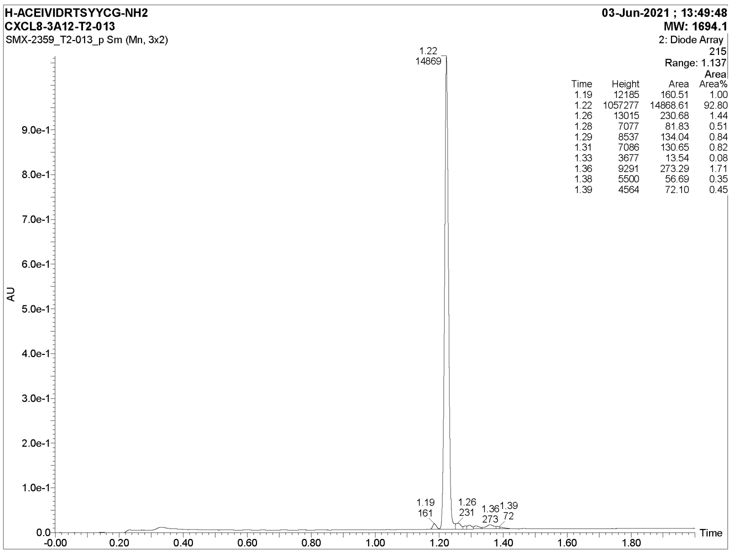

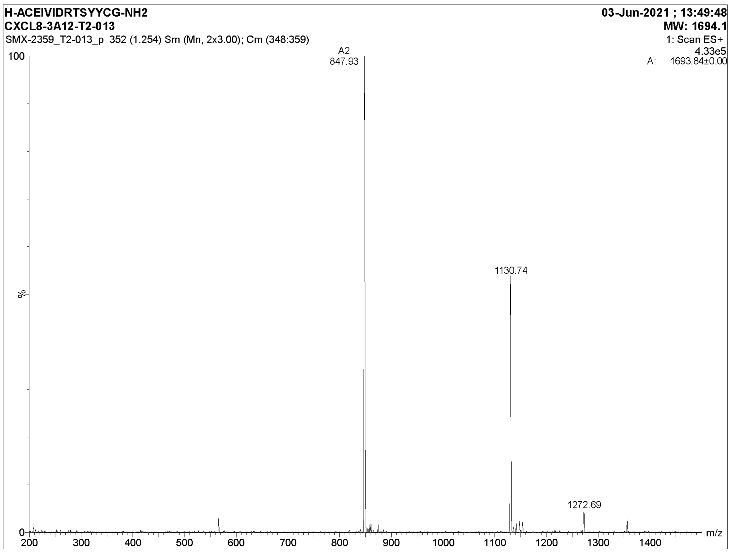

Figure S13. LC-MS analysis of L-CLIPS^{TM} peptide 3A12.

The HPLC trace (top) and ESI mass spectrum (bottom) of L-CLIPS^{TM} peptide 3A12. Observed *m/z* signals: $[M+2H]^{2+}$ - 847.9, $[2M+3H]^{3+}$ - 1130.7, $[3M+4H]^{4+}$ - 1272.7; calculated *m/z* signals: $[M+2H]^{2+}$ - 848.1, $[2M+3H]^{3+}$ - 1130.4, $[3M+4H]^{4+}$ - 1271.6.

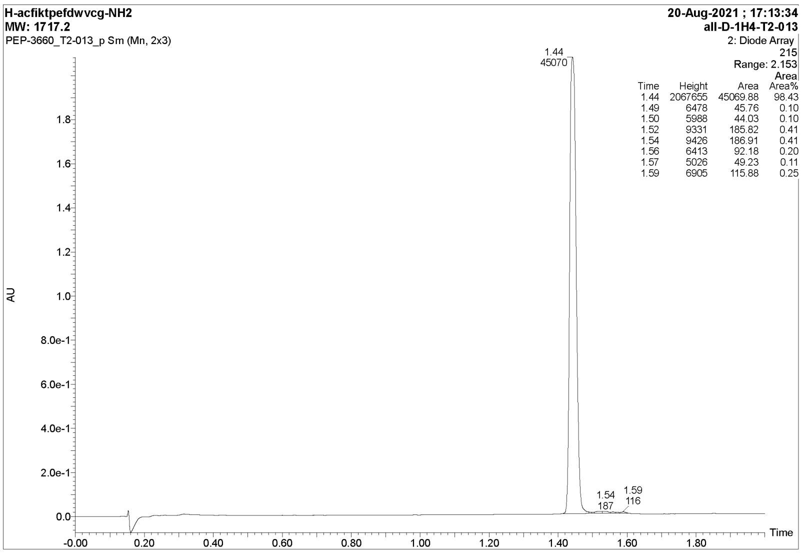

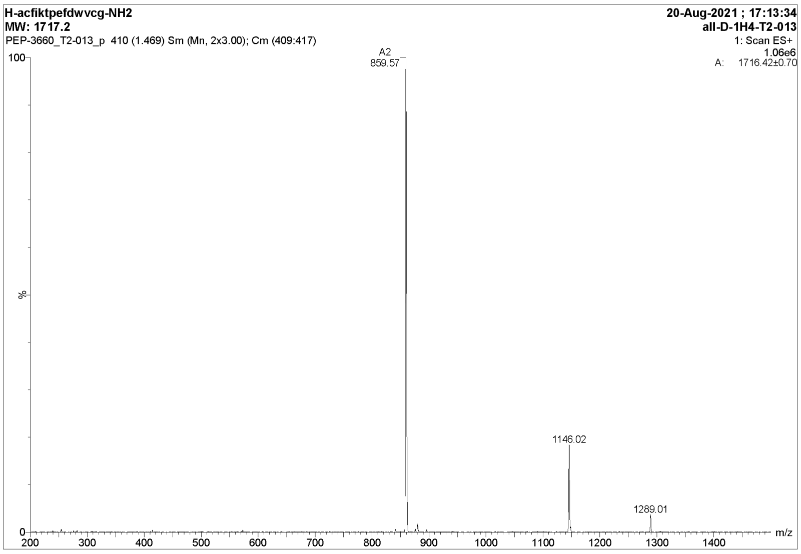

Figure S14. LC-MS analysis of D-CLIPS™ peptide 1H4

The HPLC trace (top) and ESI mass spectrum (bottom) of D-CLIPS™ peptide 1H4. Observed *m/z* signals: $[M+2H]^{2+}$ - 859.7, $[2M+3H]^{3+}$ - 1146.0, $[3M+4H]^{4+}$ - 1289.0; calculated *m/z* signals: $[M+2H]^{2+}$ - 859.6, $[2M+3H]^{3+}$ - 1145.8, $[3M+4H]^{4+}$ - 1288.9.

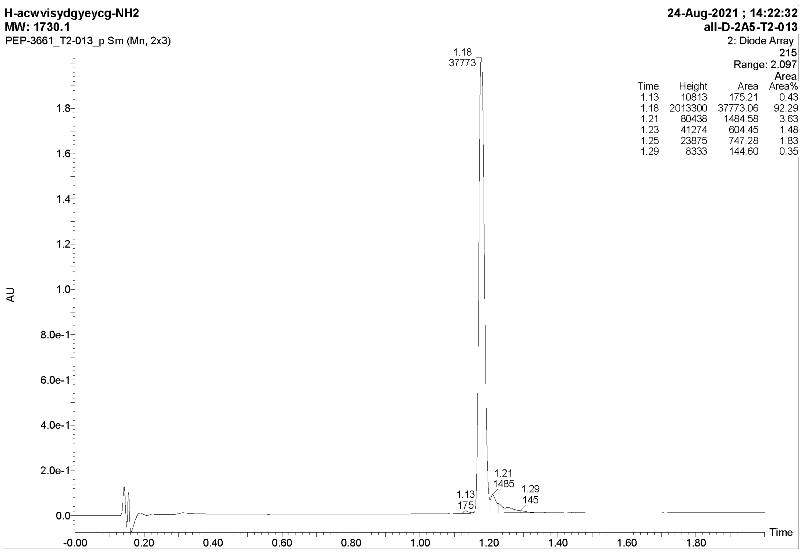

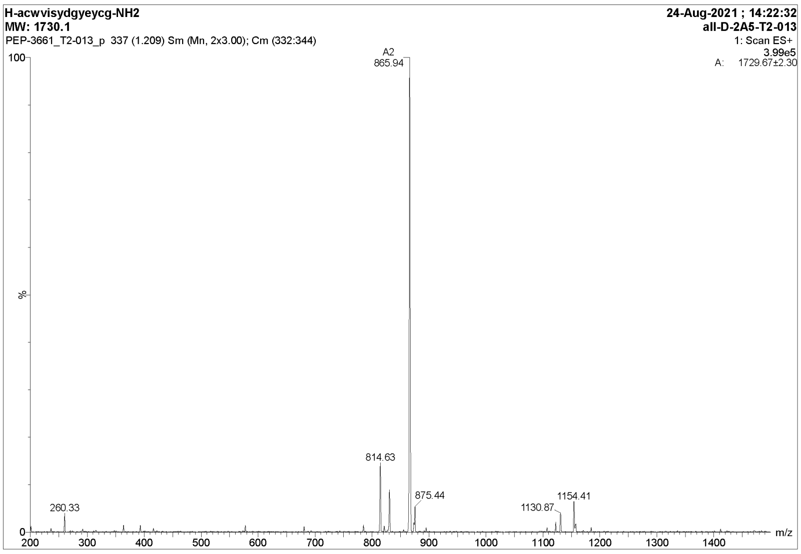

Figure S15. LC-MS analysis of D-CLIPS^{TM} peptide 2A5

The HPLC trace (top) and ESI mass spectrum (bottom) of D-CLIPS^{TM} peptide 2A5. Observed *m/z* signals: $[M+2H]^{2+}$ - 865.9, $[2M+3H]^{3+}$ - 1154.4, calculated *m/z* signals: $[M+2H]^{2+}$ - 866.1, $[2M+3H]^{3+}$ - 1154.4.

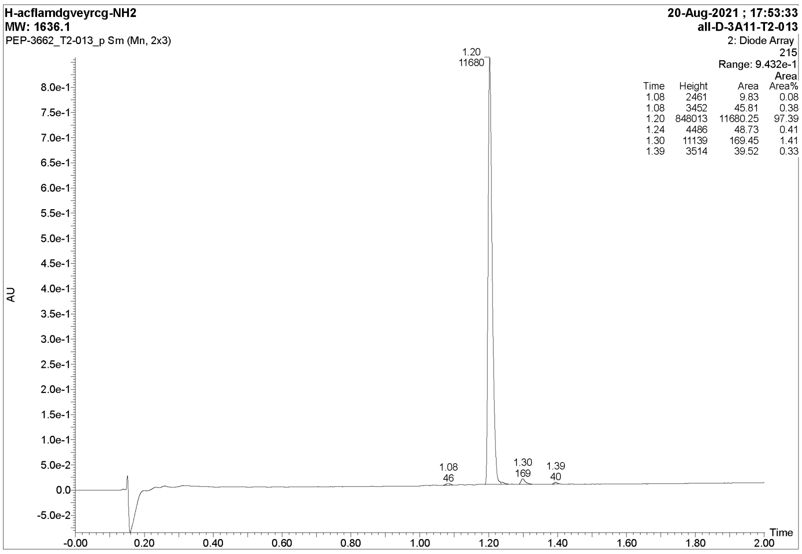

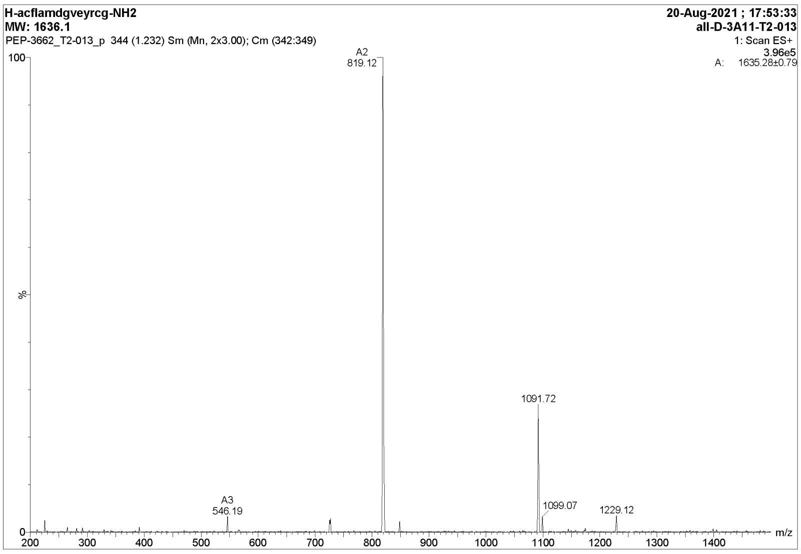

Figure S16. LC-MS analysis of D-CLIPS™ peptide 3A11.

The HPLC trace (top) and ESI mass spectrum (bottom) of D-CLIPS™ peptide 3A11. Observed *m/z* signals: $[M+2H]^{2+}$ - 819.1, $[2M+3H]^{3+}$ - 1091.7, $[3M+4H]^{4+}$ - 1229.1; calculated *m/z* signals: $[M+2H]^{2+}$ - 819.1, $[2M+3H]^{3+}$ - 1091.7, $[3M+4H]^{4+}$ - 1228.1.

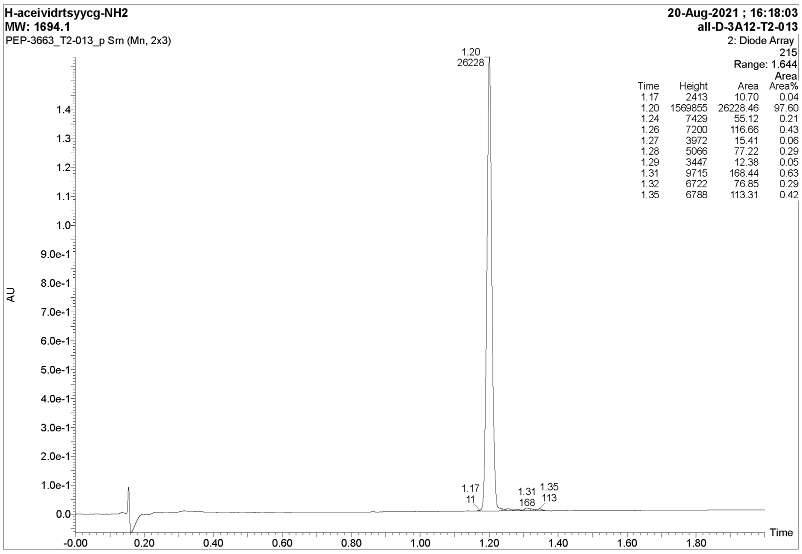

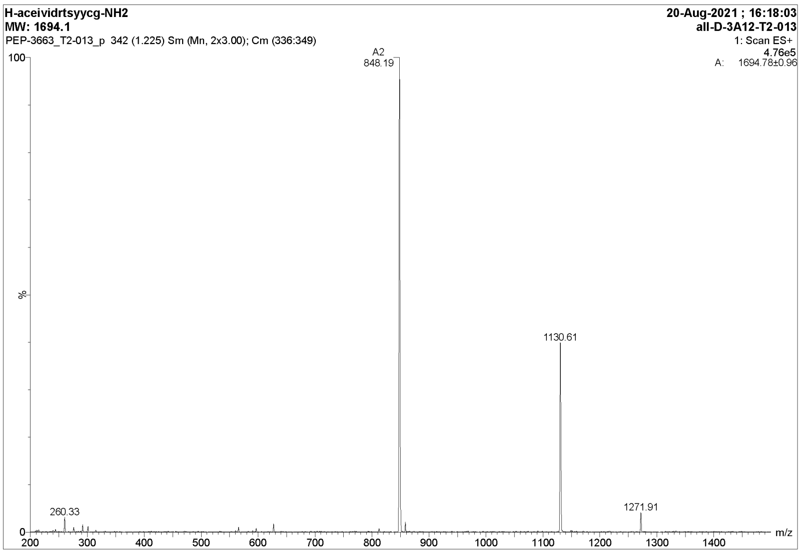

Figure S17. LC-MS analysis of D-CLIPS™ peptide 3A12.

The HPLC trace (top) and ESI mass spectrum (bottom) of D-CLIPS™ peptide 3A12 Observed *m/z* signals: $[M+2H]^{2+}$ - 848.2, $[2M+3H]^{3+}$ - 1130.6, $[3M+4H]^{4+}$ - 1271.9; calculated *m/z* signals: $[M+2H]^{2+}$ - 848.1, $[2M+3H]^{3+}$ - 1130.4, $[3M+4H]^{4+}$ - 1271.2.

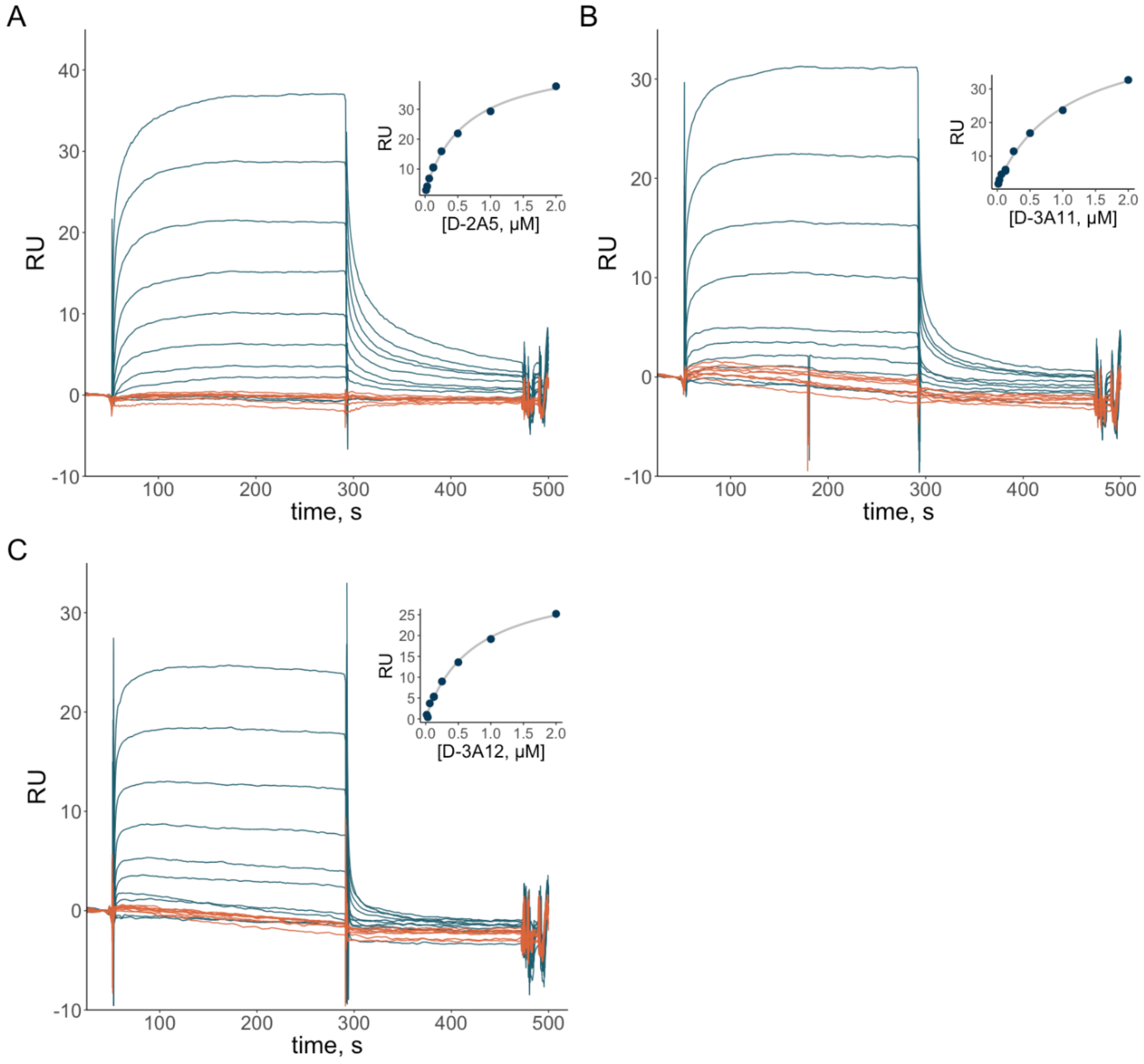

Figure S18. Binding of D-peptides to L- and D-CXCL8.
SPR biosensor analysis of interactions of D-CLIPS™ peptide 2A5 (A), D-CLIPS™ peptide 3A11 (B), and D-CLIPS™ peptide 3A12 (C) with D-CXCL8-KGG-biotin (orange) and L-CXCL8-KGG-biotin (blue) at concentrations 15 nM – 2 μM. Binding curves are shown in the inserts**.**

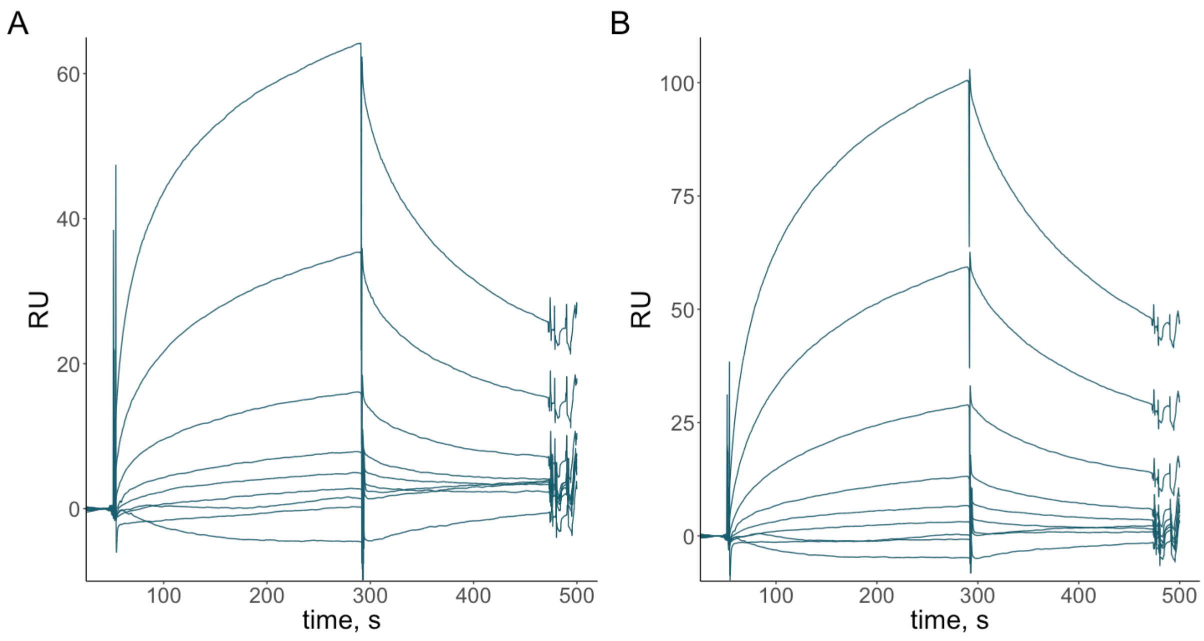

Figure S19. Binding of D-2A5 to L-CCL5 and L-CXCL4
SPR biosensor analysis of the interaction of D-2A5 at concentrations 15 nM – 2000 nM with L-CXCL4 (A) and L-CCL5 (B).

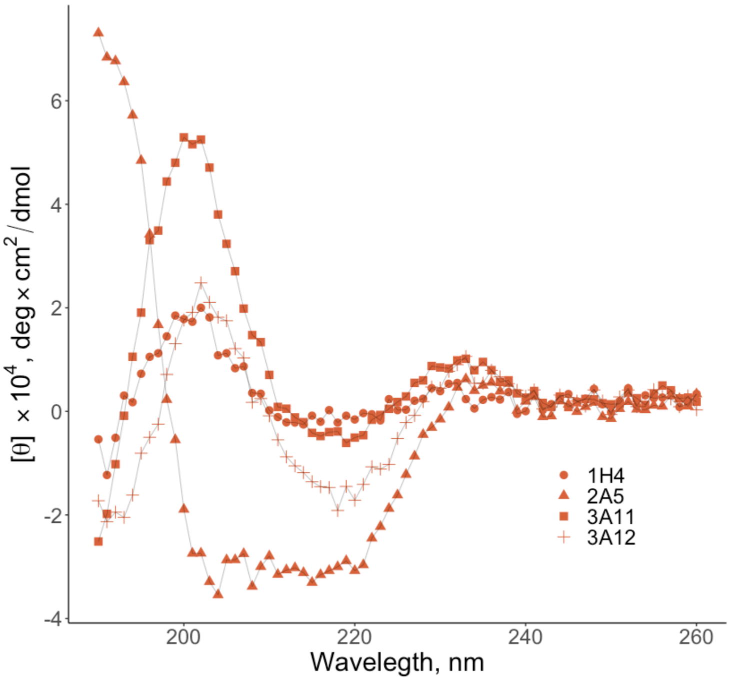

Figure S20. Far-UV CD spectra of L-CLIPS peptides. Spectra were recorded at a concentration of 0.1 mg/mL.

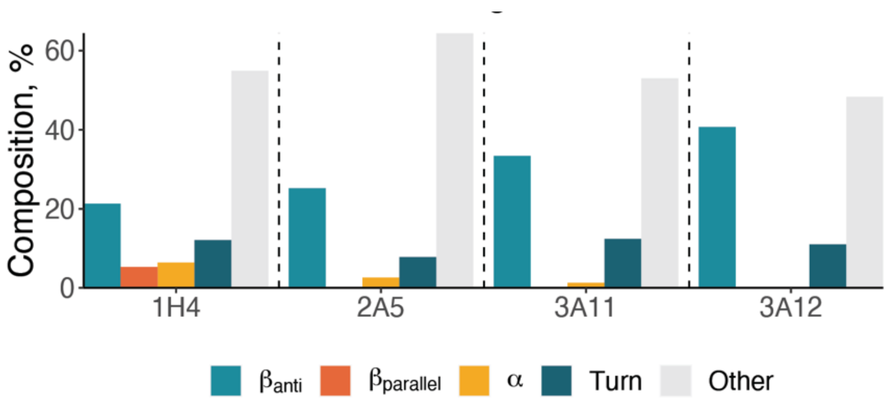

Figure S21. Secondary structure composition of CLIPSTM peptides determined by the BeStSel algorithm using CD spectra at 0.1 mg/mL of L-peptides.

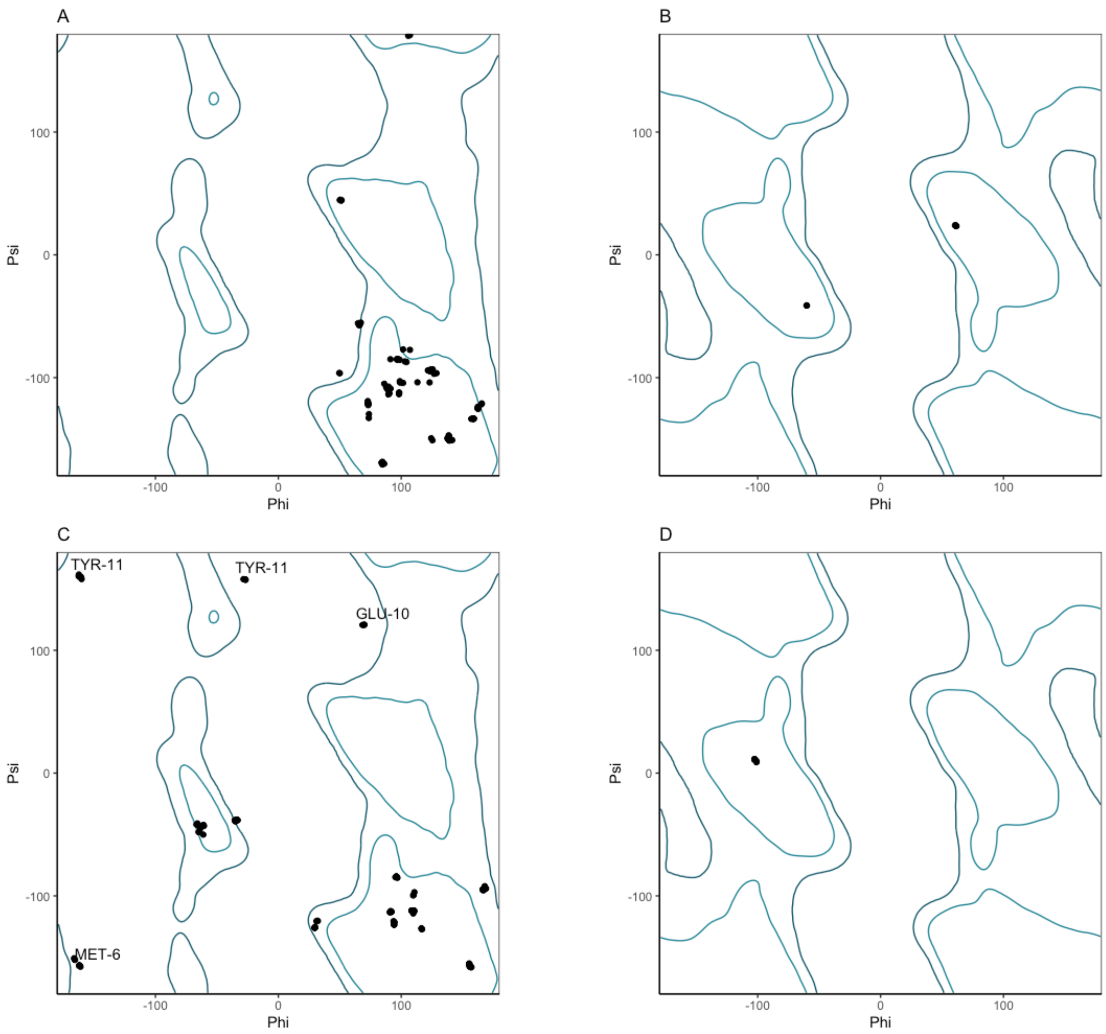

Figure S22. Ramachandran plots of calculated D-peptide models

Ramachandran plots for ϕ and ψ angles for residues of 10 lowest-energy models of D-2A5 (A – general, B – Gly) and D-3A11 (C – general, D – Gly). Outliers in D-3A11 models are captioned.

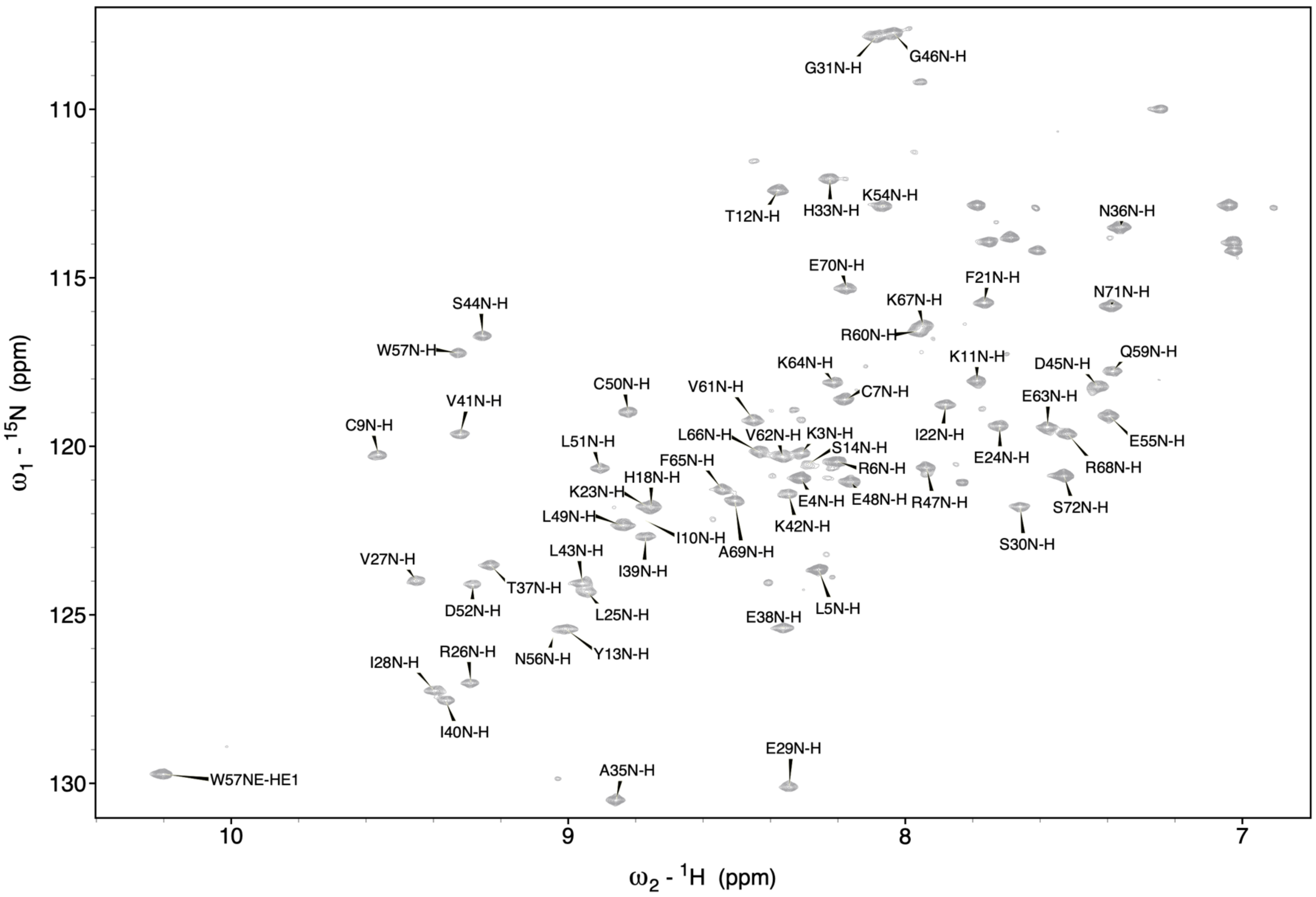

Figure S23. ^{15}N-^{1}H HSQC spectra [^{15}N, ^{13}C] L-CXCL8 dimer.

Spectra of 25 μM [^{15}N, ^{13}C] L-CXCL8 in 50 mM phosphate buffer, pH 7, 25 °C. Sidechain signals from Gln and Asn are hidden for visibility.

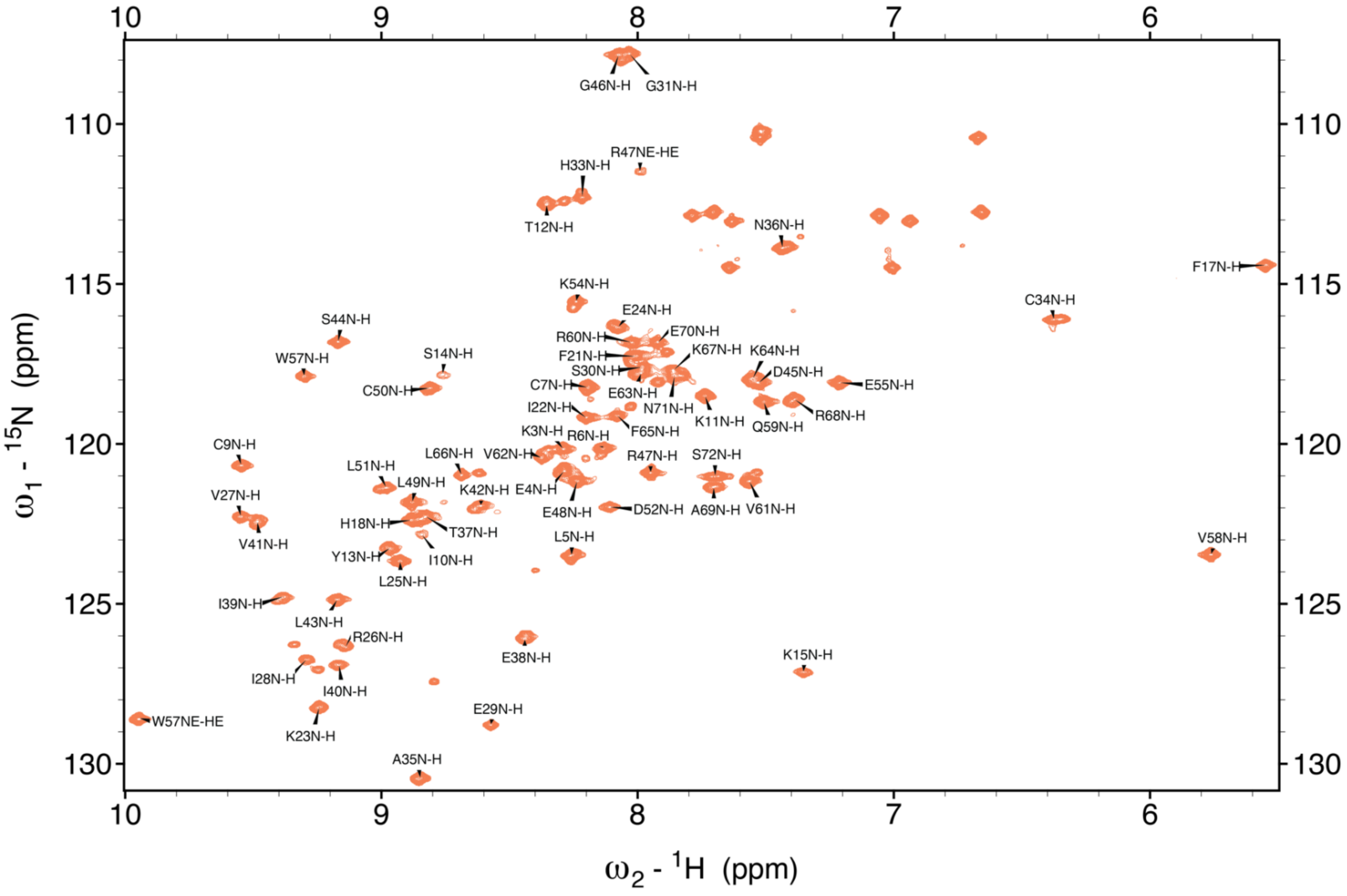

Figure S24. ^{15}N-^{1}H HSQC spectra [^{15}N, ^{13}C] L-CXCL8/D-2A5 complex.
Spectra of 59 µM [^{15}N, ^{13}C] L-CXCL8 in the presence of 300 µM D-2A5 in 50 mM phosphate buffer, 10% DMSO, pH 7, 25 °C. Sidechain signals from Gln and Asn are hidden for visibility.

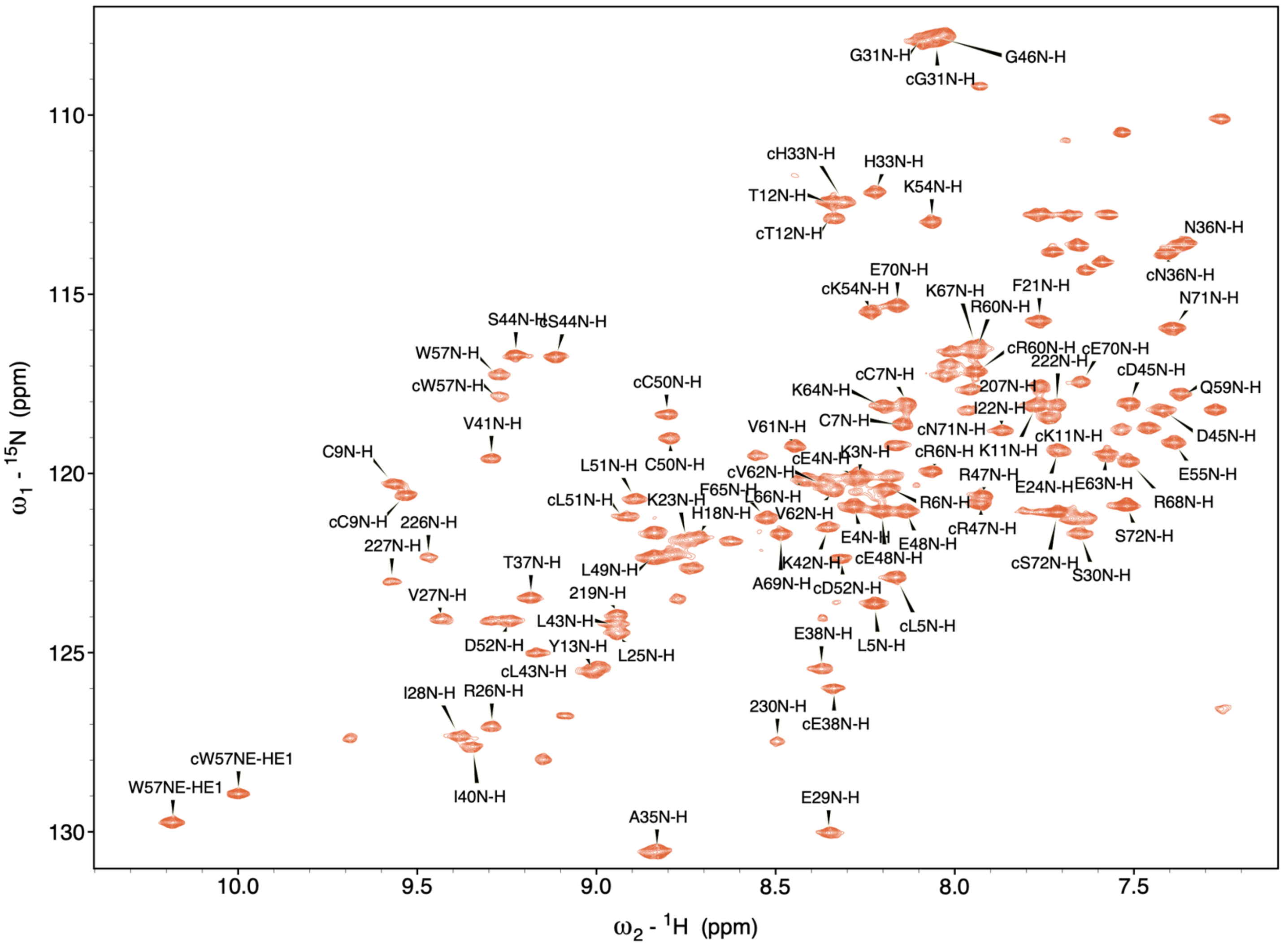

Figure S25. ^{15}N-^{1}H HSQC spectra of [^{15}N, 13C] L-CXCL8/D-3A11 complex

The spectrum of 75 µM [^{15}N, ^{13}C] L-CXCL8 in the presence of 300 µM D-3A11 in 50 mM phosphate buffer, pH 7, 25 °C. Unambiguously assigned signals from the bound L-CXCL8 are indicated with “c” and sidechain signals from Gln and Asn are hidden for visibility.
